# Baseline and interim ^18^F-FDG PET/CT metabolic parameters predict the efficacy and survival in patients with diffuse large B-cell lymphoma

**DOI:** 10.3389/fonc.2024.1395824

**Published:** 2024-10-07

**Authors:** Chengcheng Liao, Qifeng Deng, Lin Zeng, Baoping Guo, Zhe Li, Da Zhou, Qing Ke, Mingyue Wang, Mei Huang, Xiaohong Tan, Hong Cen

**Affiliations:** ^1^ Department of Hematology/Oncology, Guangxi Medical University Cancer Hospital, Nanning, Guangxi, China; ^2^ State Key Laboratory of Targeting Oncology, Guangxi Medical University, Nanning, Guangxi, China; ^3^ Oncology Prevention and Control Center, Guigang People’s Hospital, Guigang, Guangxi, China; ^4^ Department of Thyroid and Breast Surgery, The First Affiliated Hospital of Guangxi University of Chinese Medicine, Guangxi, Nanning, China; ^5^ College of Oncology, Guangxi Medical University, Nanning, China

**Keywords:** diffuse large B-cell lymphoma, ^18^F-FDG PET/CT, total lesion glycolysis, metabolic tumor volume, prognostic factors analysis

## Abstract

**Introduction:**

The prognostic value of 18F-FDG PET/CT metabolic parameters, such as metabolic tumor volume (MTV) and total lesion glycolysis (TLG), in diffuse large B-cell lymphoma (DLBCL) remains inadequately explored. This study aims to assess the correlation between these parameters and patient outcomes.

**Methods:**

A cohort of 156 DLBCL patients underwent 18F-FDG PET/CT imaging at baseline and after 3-4 cycles of R-CHOP or CHOP-like regimen. The third quartiles of liver uptake values were used as thresholds for calculating MTV and TLG. Patient outcomes were analyzed based on Ann Arbor staging and the 5-PS score. A nomogram was developed to predict overall survival (OS).

**Results:**

Patients with low baseline TLG exhibited significantly better outcomes compared to those with high baseline TLG in both Ann Arbor stages I-II and III-IV (1-year PFS: 78.9% vs. 40%, p=0.016; OS: 94.7% vs. 40%, p=0.005 for stage I-II; 1-year PFS: 74.1% vs. 46.8%, p=0.014; OS: 85.4% vs. 71.8%, p=0.007 for stage III-IV). In interim PET/CT patients with a 5-PS score >3, the high ΔTLG group had superior prognosis (1-year PFS: 82.3% vs. 35.7%, p=0.003; OS: 88.2% vs. 85.7%, p=0.003). The nomogram achieved a C-index of 0.9 for OS prediction.

**Discussion:**

The findings suggest that baseline TLG is a robust prognostic indicator for patients with DLBCL, particularly in early stages, while ΔTLG effectively distinguishes those with favorable outcomes in higher-risk groups. These metabolic parameters from 18F-FDG PET/CT could enhance treatment decision-making and patient management strategies.

## 1 Introduction

Diffuse large B-cell lymphoma (DLBCL) is the most common subtype of aggressive non-Hodgkin lymphoma (NHL), accounts for approximately 30% of all NHL. Most patients with DLBCL can achieve a cure through chemotherapy and targeted therapies, and the widely adopted first-line treatment include the rituximab, cyclophosphamide, doxorubicin, vincristine, and prednisone (R-CHOP) regimen. With R-CHOP or modified R-CHOP regimens, over 50% of patients attain complete remission, but up to one-third may experience relapse or refractory disease (1, 2). The main reason for this is tumor drug resistance, and it has been shown that Long non-coding RNA SNHG17 plays an important role in the progression of DLBCL (3, 4). A previous study has indicated that patients undergoing autologous stem cell transplantation had a 1-year overall survival rate (OS) of only 41.6% (5). Therefore, the early identification of patients with poor prognosis or insensitivity to first-line treatment is crucial for improving outcomes (6).

Many studies have attempted to use noninvasive tests to predict the prognosis of patients with DLBCL; for example, it has been suggested that pelvic MRI is effective in detecting bone marrow involvement (BMinv) in patients with DLBCL and that it may ultimately be used to improve the accuracy of clinical staging, guide the treatment of patients, and assess prognosis (7). Currently, ^18^F-FDG PET/CT is a common imaging modality for the diagnosis and treatment of DLBCL and plays a significant role in staging, treatment monitoring, and treatment response assessment (8, 9). However, the value of interim PET/CT for mid-term efficacy and prognostic assessment in patients with DLBCL remains controversial, with no established gold standard for evaluation. The First International Lymphoma PET/CT Workshop, held in Deauville, France in 2009, recommended the use of the Deauville five-point scale (5-PS) to assess different responses to lymphoma treatment at mid-term and post-treatment (10). The 5-PS scoring is assigned based on the Standardized Uptake Value max (SUVmax) of the lesion with the highest uptake, as follows: 1 point: no uptake; 2 points: uptake≤mediastinum; 3 points: mediastinum<uptake≤liver; 4 points: uptake moderately higher than liver; 5 points: uptake markedly higher than liver and/or new lesions; X: new areas of uptake unlikely to be related to lymphoma. A Deauville score of 1–3 is considered complete metabolic remission (CMR), and a score of 4–5 is categorized as follows: if the uptake is lower than the baseline PET/CT, it is considered a partial metabolic response (PMR); if there is no significant change in uptake compared to the baseline PET/CT and no new or progressing lesions, it is classified as no metabolic response (NMR); if the uptake is higher than the baseline PET/CT and/or new lesions appear, it is considered a progressive metabolic disease (PMD) (10, 11). The 5-PS score does not require complex calculations, such as metabolic tumor volume (MTV) and total lesion glycolysis (TLG), and reduces the impact of different PET/CT equipment. The reports issued by different hospitals can be efficiently compared. Owing to its simplicity and efficiency, the PET/CT performed in the interim during chemotherapy has been widely investigated for response-adapted therapy in Hodgkin’s lymphoma (HL), DLBCL, and other subsets of NHL (12).

Commonly used PET/CT metabolic parameters in clinical practice include the MTV, TLG, SUVmax, and Standardized Uptake Value mean (SUVmean). SUVmax, which represents the highest uptake intensity within a Volume of Interest (VOI), is widely used in pre-treatment assessment, mid-term efficacy evaluation, and post-treatment efficacy assessment of lymphoma treatment. However, SUVmax was measured at the site of highest uptake, reflecting the metabolic intensity of the most active tumor cells. Consequently, factors, such as different PET/CT devices, tumor heterogeneity, image algorithms, and scan intervals, can significantly affect the SUVmax (5, 13, 14). In recent years, with the widespread clinical application of ^18^F-FDG PET/CT, an increasing number of studies have shown that using MTV and TLG for the prognostic evaluation of patients with lymphoma can improve the accuracy of ^18^F-FDG PET/CT in predicting lymphoma prognosis (15–17).

Currently, there is no gold standard for determining the threshold for the MTV, and different thresholds may yield significantly different MTV and TLG measurements for the same patient. Three common methods have previously been used to determine the marginal threshold for the MTV. One method uses an absolute cutoff value of 2.5 as the SUV threshold, defining tissues with an absolute SUV value greater than 2.5 as tumor lesions (18–20). However, many factors can significantly affect the absolute SUV cutoff value, such as the time interval between tracer injection and scanning, different PET equipment, and injection malfunctions, rendering the use of the absolute SUV cutoff value as a threshold for measuring the MTV inaccurate. The second method uses a certain percentage of SUVmax in the most metabolically active lesion to determine MTV, commonly ranging from 25% to 75% (21–23). However, owing to the variations in uptake in different lesions and pathological subtypes, the ideal percentage may differ, and there is currently no unified standard for the best SUVmax ratio for measuring the MTV. When a higher percentage of SUVmax is used, there is a risk of underestimating tumor volume, whereas a lower percentage may lead to an overestimation of tumor volume. The third method calculates the patient’s own liver SUVmean and adds two to three times the standard deviation (SD) to obtain the threshold (24, 25). This method can significantly reduce the impact of different PET/CT technologies and subjective factors. The Positron Emission Tomography Response Criteria in Solid Tumors (PERCIST) also recommends using MTV and TLG to predict patient prognosis (26). However, the predictive abilities of these parameters vary across studies, and there is no unified standard for measuring the MTV or TLG.

In this study, a PET/CT Lesion Quantifier, which significantly reduced the calculation complexity and improved the calculation speed, was used to calculate the MTV and TLG. We investigated the relationship between baseline MTV, TLG, and interim ΔMTV and ΔTLG(after 3-4 cycles of treatment) with the prognosis of patients with DLBCL and attempted to compare with the 5-PS score. This study aimed to explore which parameters, MTV or TLG, are more suitable for predicting the prognosis of patients with DLBCL and investigate the value of interim PET/CT for mid-term efficacy and prognosis assessment in patients with DLBCL. Inspired by the Deauville score, which uses mediastinal and liver uptake values as scoring criteria, we used the mean, third quartile, maximum, 1.5 × the mean, and two times the mean of the mediastinal and liver uptake values as thresholds to measure the MTV and TLG, respectively. After screening, we chose the third quartile of liver uptake values as the threshold for the measurement of MTV and TLG in patients for the next step of the study (the specific screening process is shown in the **Supplementary Material**).

## 2 Patients and methods

### 2.1 Patient population

This study included 156 patients diagnosed with diffuse large B-cell lymphoma at Guangxi Medical University Cancer Hospital between December 2017 and July 2021. The inclusion and exclusion criteria were as follows.

The inclusion Criteria: 1) Age ≥18 years old; 2) Pathological results confirmed as diffuse large B-cell lymphoma; 3) Complete PET/CT examination in our hospital before anti-tumor treatment; 4) R-CHOP or modified R-CHOP was used after the diagnosis was confirmed Program treatment.

The exclusion criteria: 1) Pregnant and lactating women; 2) Unable to complete PET/CT examination due to claustrophobia or other reasons; 3) Due to lack of original PET/CT images or reports; 4) Have a history of comorbidity with other malignant tumors. 5) Disappearance of primary lesions due to antitumor treatment before completing baseline PET/CT.

### 2.2 Patient characteristics

Among the 156 eligible patients, 74 patients (47.4%) had 0-1 extranodal site and 82 patients (52.6%) had two or more extranodal sites. According to the Ann Arbor stage, 10 patients (6.4%) had stage I, 52 patients (33.3%) had stage II, 31 patients (19.9%) had stage III, and 63 patients (40.4%) had stage IV. We provide specific data on gender, age, ECOG-PS, β2-microglobulin, Ki-67 index, blood glucose, and LDH in **Table 1**.

**Table 1:** Clinical features of the patient.

clinical features	Value/percentage
Total number of patients	156 (100%)
Genders	
male	90 (7.6%)
female	66 (42.4%)
Age	
≤ 60	96 (61.5%)
> 60	60 (38.5%)
ECOG-PS	
0-1	135 (86.5%)
≥ 2	21 (13.5%)
Extranodal site	
0-1	74 (47.4%)
≥ 2	82 (52.6%)
Ann Arbor Stage	
I-II	62 (39.7%)
III-IV	94 (60.3%)
β2-Microglobulin	3.03±2.27 (mg/L)
Ki-67 Index	72.0±19 (%)
Treatment Regimen	
R-CHOP	124 (79.50%)
R2-CHOP	23 (14.74%)
R-miniCHOP	4 (2.56%)
DA-E-POCH-R	4 (2.56%)
R-CHOEP	1 (0.64%)

The median follow-up period was 26 months. The 1-year overall survival (OS) rates were 83.9%, and the 1-year progression-free survival (PFS) rate was 69.2%. Out of 156 patients, 124 patients used the R-CHOP regimen and the rest used the R2-CHOP, R-mini CHOP, DA-E-POCH-R, and R-CHOEP regimens. Detailed information is provided in **Table 1**.

### 2.3 Statistical analysis

Data analysis and graphical representation were conducted using R software (version 4.2.1). Cox proportional hazard models were used for both univariate and multivariate regression analyses. These analyses aimed to assess the relationships among each clinical prognostic factor, ^18^F-FDG PET/CT metabolic parameters, patient PFS, and OS. Survival analysis was performed using the Kaplan–Meier method. Statistical significance was set at p < 0.05.

### 2.4 Acquisition of metabolic parameters in ^18^F-FDG PET/CT

**Figure 1:**
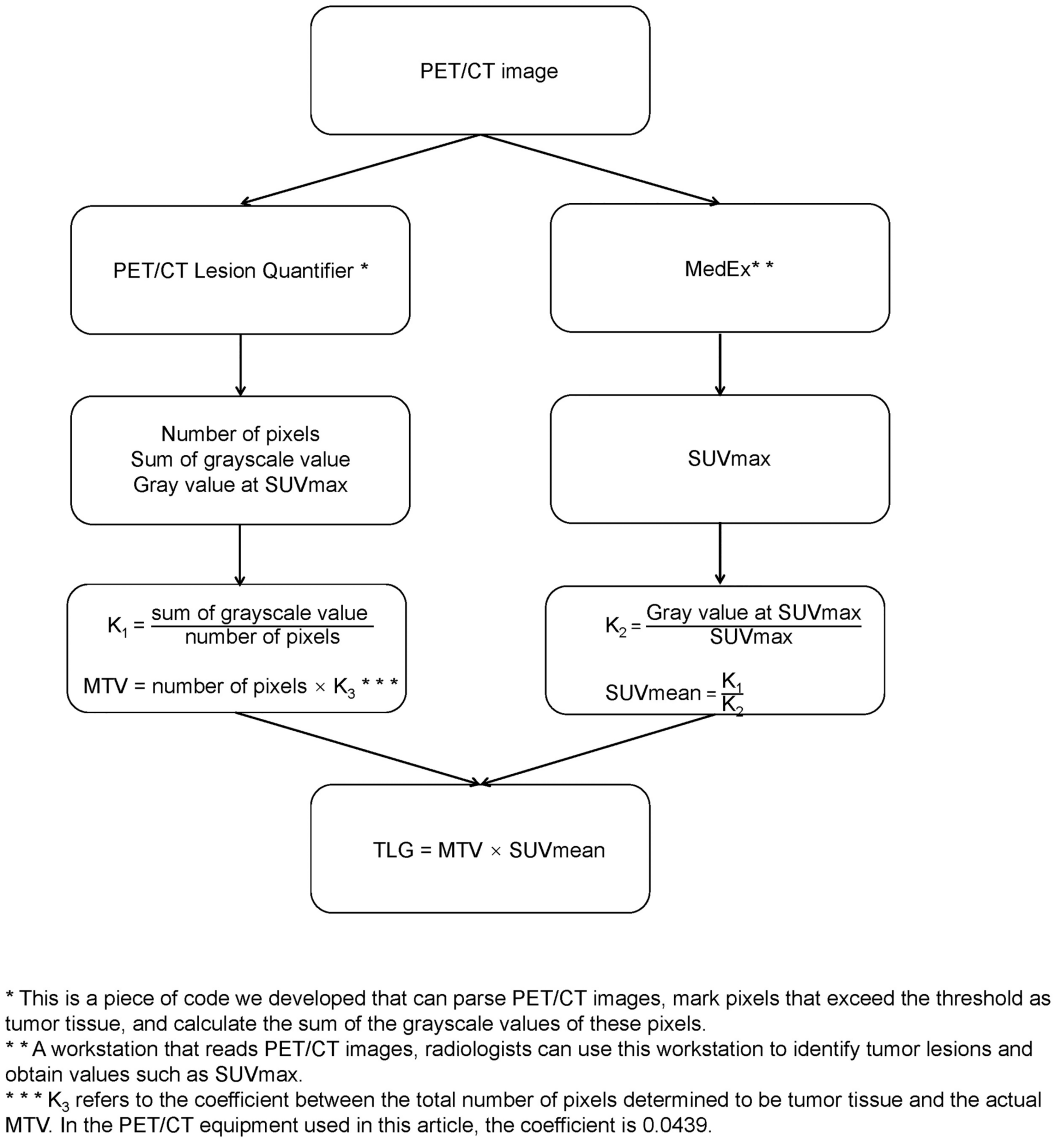
Calculation process of MTV, TLG.

The preparation, scan parameters, and image processing for ^18^F-FDG PET/CT include the following: (1) fasting for 6 h before the examination, abstaining from glucose infusion, and measuring fasting blood glucose below 150 mg/dL; (2) administration of the radiotracer, ^18^F-FDG (dose: 5.55 MBq/Kg), followed by rest in a quiet, dimly lit environment with water intake of 500–1000 ml, and scanning after 1 h; (3) image acquisition using the GE Discovery 710PET/CT, scanning from the top of the skull to the level of the proximal femur; (4) CT acquisition parameters for the 64-slice CT scanner are as follows: tube voltage 120 kV, tube current 110 mAs; rotation time 0.5 s, slice thickness 3.3 mm, pitch 0.8, matrix 512×512; and (5) attenuation correction based on CT, image reconstruction using the Ordered Subset Expectation Maximization (OSEM) algorithm, and fusion of PET and CT images. Two experienced radiologists determined the maximum standardized uptake value (SUVmax) in the PET/CT report using a MedEx workstation. The radiologists were blinded to the patients’ clinical outcomes and treatment plans. The original PET/CT images were then imported into a PET/CT Lesion Quantifier. Combined with the PET/CT report provided by the radiologist, the layer with the highest uptake intensity was selected to obtain the gray value at SUVmax. The liver uptake levels of the patients were first identified on the PET/CT images, and the third quartile of the uptake values in the obtained range was set as the threshold. The PET/CT Lesion Quantifier automatically identified points on the PET/CT images with uptake values higher than this threshold and classified them as tumor lesions. The total number of pixels representing the tumor tissue can be obtained by adding all pixels that are judged to be tumor tissues. The sum of the gray values of the above pixels represents the intensity of tumor metabolism. To minimize the effect of body weight, the values of the sum of pixel volumes and gray values were corrected by dividing them by the patient’s weight in kilograms before further calculation. Each layer of the PET/CT image had an actual size of 70 × 70 cm^2^, with a thickness of 0.33 cm, resulting in an actual volume of 1617 cm^3^ for each layer. The PET/CT Lesion Quantifier displayed each layer as a 192×192 matrix, with 36864 points for each layer. Therefore, the actual volume of each point in the matrix was 0.0439 cm^3^. Based on the total number of lesion points obtained, the actual MTV was calculated as follows: MTV (cm3) = total number of pixels representing the lesion × 0.0439. Using the formula SUVmean × MTV = TLG, the actual TLG was calculated from the known MTV and SUVmean. Considering that the uptake value of glucose by the brain is signifi-cantly higher than that of normal tissues, we removed the portion of the brain in the PETCT images before calculating the MTV and TLG.In the sample of this study, it takes 119 ± 24 seconds to calculate the MTV and TLG of a patient.The calculation process is illustrated in **Figure 1**.

Some studies have reported that interim PET/CT analysis after 3–4 chemotherapy cycles can predict disease prognosis (27). PET/CT was completed before treatment as baseline PET/CT (PET/CT0), and PET/CT was completed after 3–4 cycles of treatment as interim PET/CT (PET/CT1). The same method was used to calculate MTV and TLG for interim PET/CT. The equations for calculating ΔMTV and ΔTLG are as follows:


$$\text{ΔMTV}=\frac{\text{MTV (PET}/\text{CT}0)-\text{ MTV (PET}/\text{CT}1)}{\text{ MTV (PET}/\text{CT }0)}\times 100\%$$

$$\text{ΔTLG}=\frac{\text{TLG (PET}/\text{CT}0)-\text{ TLG (PET}/\text{CT}1)}{\text{ TLG (PET}/\text{CT }0)}\times 100\%$$

## 3 Results

### 3.1 Baseline ^18^F-FDG PET/CT

#### 3.1.1 Univariate and multivariate Cox proportional hazards model analyses

The results of the univariate Cox proportional hazards model analysis revealed a significant correlation between MTV and patients’ progression-free survival (PFS) (p=0.003) as well as overall survival (OS) (p=0.0007). Similarly, TLG was found to be significantly correlated with PFS (p=0.0006) and OS (p=0.0002). Clinical factors, such as Ann Arbor stage, extranodal sites, β2-microglobulin, and ECOG performance status, were also significantly associated with patients’ prognosis. However, SUVmax was not significantly correlated with either PFS or OS. Specific values are shown in **Tables 1**–**3**.

**Table 2:** Univariate Cox proportional hazards model analysis of MTV, TLG, clinical factors and patients' PFS.

	HR(95%CI)	p-value
Ann Arbor Stage	2.04 (1.13-3.68)	0.018
ECOG-PS	3.05 (1.66-5.61)	0.0003
Extranodal sites	1.15 (1.06-1.25)	0.0008
β2-microglobulin	1.17 (1.09-1.26)	<0.0001
SUVmax	0.98 (0.95-1.01)	0.269
MTV	1.32 (1.10-1.59)	0.003
TLG	1.68 (1.25-2.26)	0.0006

**Table 3:** Univariate Cox proportional hazards model analysis of MTV, TLG, clinical factors and patients' OS.

	HR(95%CI)	p-value
Ann Arbor Stage	2.46 (1.21-5.02)	0.012
ECOG-PS	4.83 (2.48-9.41)	<0.0001
Extranodal sites	1.23 (1.12-1.34)	<0.0001
β2-microglobulin	1.17 (1.09-1.25)	<0.0001
SUVmax	0.98 (0.95-1.02)	0.478
MTV	1.40 (1.15-1.69)	0.0007
TLG	1.83 (1.33-2.50)	0.0002

Based on the results of univariate Cox proportional hazards model analysis, factors that were significantly correlated with both PFS and OS, including MTV, TLG, and clinical factors, were subjected to multivariate Cox proportional hazards model analysis. The results indicate that the MTV was not an independent predictor of PFS (p=0.059) or OS (p=0.068). In contrast, the TLG was an independent predictor of PFS (P =0.016) and OS (P =0.015). The specific values are listed in **Tables 4** and **5**.

**Table 4:** Multivariate Cox proportional hazards model analysis of MTV and patients' PFS and OS.

	PFS		OS	
	HR (95%CI)	p-value	HR (95%CI)	p-value
Ann Arbor Staging	1.23 (0.63-2.39)	0.533	1.24 (0.56-2.77)	0.586
ECOG-PS	1.37 (0.62-3.02)	0.433	1.70 (0.68-1.24)	0.252
Extranodal sites	1.07 (0.97-1.18)	0.138	1.13 (1.02-1.25)	0.017
β2-microglobulin	1.14 (1.03-2.12)	0.005	1.12 (1.02-1.23)	0.016
MTV	1.21 (0.99-1.47)	0.059	1.00 (0.99-1.00)	0.068

**Table 5:** Multivariate Cox proportional hazards model analysis of TLG and patients' PFS and OS.

	PFS		OS	
	HR (95%CI)	p-value	HR (95%CI)	p-value
Ann Arbor Staging	0.87 (0.44-1.71)	0.706	1.11 (0.49-2.49)	0.803
ECOG-PS	1.37 (0.64-2.94)	0.405	1.77 (0.76-4.15)	0.183
Extranodal sites	1.06 (0.97-1.17)	0.168	1.12 (1.01-1.24)	0.025
β2-microglobulin	1.16 (1.03-1.24)	0.007	1.11 (1.01-1.22)	0.018
TLG	1.24 (1.04-1.48)	0.016	1.29 (1.05-1.59)	0.015

#### 3.1.2 Kaplan–Meier curves plotted based on TLG grouping

The optimal cut-off value for TLG, which was determined by the receiver operating characteristic (ROC) curve(We use the Youden’s index to calculate the optimal cutoff value, where the maximum value of the Youden’s index corresponds to the optimal diagnostic threshold for the method, i.e., the cutoff value), was 435.056. At this cutoff value, the area under the curve (AUC) was 0.678. Patients were then categorized into two groups based on this optimal cutoff: TLG≥435.056 group and TLG<435.056 group. In this group, the specificity was 85.1% and the sensitivity was 47.6%. Subsequently, patients were stratified according to the optimal cutoff value, and Kaplan–Meier survival curves were generated.

**Figure 2:**
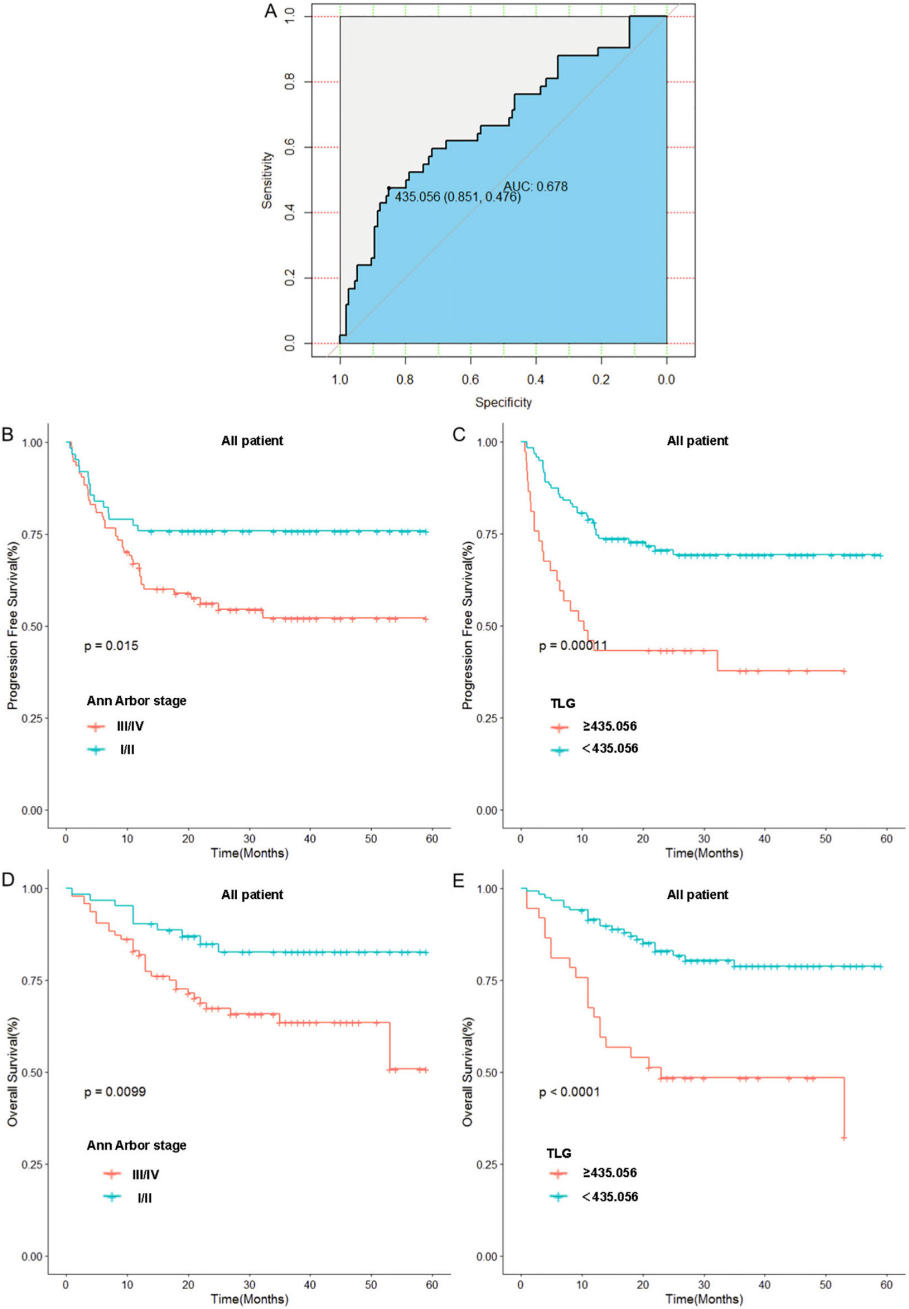
Relationship between Ann Arbor stage, baseline TLG and patients’ prognosis: **(A)** Receiver operating curve of TLG. **(B)** Relationship between Ann Arbor stage and patients’ PFS. **(C)** Relationship between TLG and patients’ PFS. **(D)** Relationship between Ann Arbor stage and patients’ OS. **(E)** Relationship between TLG and patients’ OS.

In contrast to Ann Arbor staging, we categorized patients into stage I/II and III/IV groups based on the Ann Arbor staging system and plotted Kaplan–Meier survival curves. The results indicated that the 1-year progression-free survival (PFS) rate in the I/II stage group was significantly higher than that in the III/IV stage group (75.8% vs. 64.8%, p=0.015). Low TLG group had a significantly higher 1-year PFS rate compared with the high TLG group (76.4% vs. 45.9%, p=0.0001). Similarly, the 1-year overall survival (OS) rate in the I/II stage group was significantly higher than that in the III/IV stage group (90.3% vs. 80.8%, p=0.009). Low TLG group exhibited a significantly higher 1-year OS rate than the high TLG group (89.9% vs. 67.5%, p<0.0001). Moreover, TLG grouping appears to have advantages over the Ann Arbor stage. **Figure 2** illustrates the associated Kaplan–Meier survival curves. In view of the significant correlation between MTV and patient prognosis in the univariate Cox proportional hazards model analysis, we divided the patients into the MTV ≥ 17.468 group and the MTV < 17.468 group and drew the Kaplan–Meier curve. These graphs are shown in **Supplementary Figure S1**.

**Figure 3:**
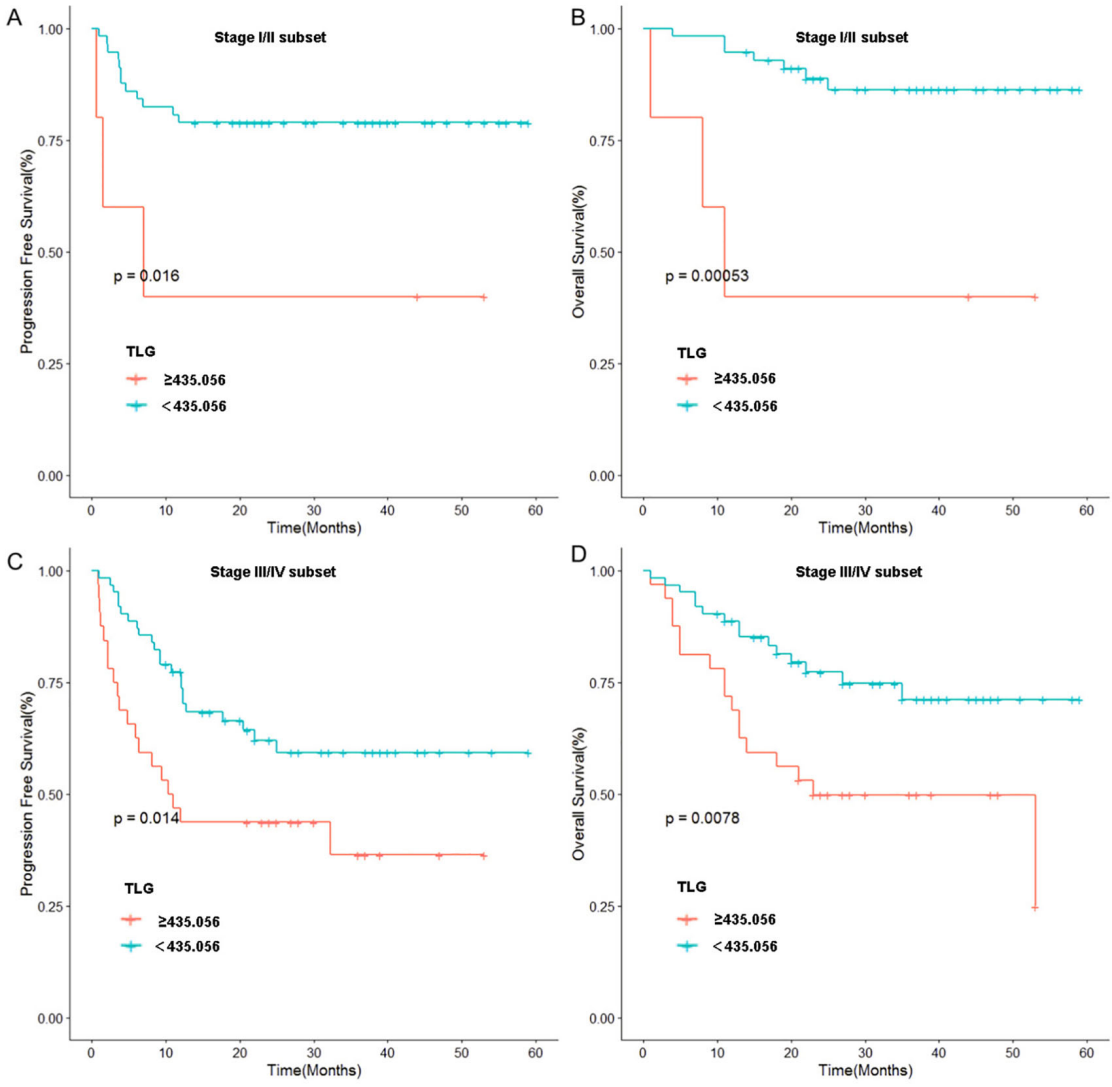
Stratified analysis of Ann Arbor stage: **(A)** Relationship between TLG and PFS in patients of stage I and II. **(B)** Relationship between TLG and OS in patients of stage I and II. **(C)** Relationship between TLG and PFS in patients of stage III and IV. **(D)** Relationship between TLG and OS in patients of stage III and IV.

To further validate these results, we used TLG to regroup the patients in Ann Arbor stages I-II and III-IV, and the results indicated that in patients with stages I-II, a significant difference was noted in prognosis between the high and low TLG groups (PFS: p=0.026; OS: p=0.003). Similarly, there was a significant difference in the prognosis between the high and low TLG groups (PFS: p=0.016; OS: p=0.013) in patients with stage III-IV disease. The specific values are listed in **Tables 6**. Therefore, Kaplan–Meier survival curves were respectively plotted for patients with stages I-II and III-IV based on TLG grouping, and the results showed that in patients with Ann Arbor stage I/II, the 1-year PFS and 1-year OS rates of the low TLG group were significantly higher than those of the high TLG group (PFS: 78.9% vs. 40%, p=0.016; OS: 94.7% vs. 40%, p=0.005). In patients with Ann Arbor stage III/IV, the 1-year PFS and 1-year OS rates of the low TLG group were significantly higher than those of the high TLG group (PFS: 74.1% vs. 46.8%, p=0.014; OS: 85.4% vs. 71.8%, p=0.007). **Figure 3** illustrates the associated Kaplan–Meier survival curves.

**Table 6:** Univariate Cox proportional hazards model analysis of TLG in patients with stages I and II or III and IV.

Ann Arbor staging	PFS		OS	
	HR (95%CI)	p-value	HR (95%CI)	p-value
I-II	4.19 (1.18-14.91)	0.026	7.87 (2.01-30.73)	0.003
III-IV	2.11 (1.15-3.87)	0.016	2.49 (1.24-4.99)	0.013

### 3.2 Interim ^18^F-FDG PET/CT

#### 3.2.1 Univariate and multivariate Cox proportional hazards model analyses

Among the 156 patients, 62 completed the interim PET/CT examination. Using the same method, the MTV and TLG of each patient were calculated for interim PET/CT, and the relationships between ΔMTV, ΔTLG, and patient prognosis were analyzed. Univariate Cox proportional hazards model analysis showed that ΔMTV was not correlated with PFS (p=0.820) or OS (p=0.281), whereas ΔTLG was significantly correlated with PFS (p<0.0001) and OS (p<0.0001). The results of the multivariate Cox proportional hazards model analysis indicated that ΔTLG is an independent predictor of DLBCL patients’ PFS (p<0.0001) and OS (p=0.0006). The specific values are listed in **Tables 7** and **8**.

**Table 7:** Univariate Cox proportional hazards model analysis of ΔMTV, ΔTLG, Clinical Factors and patients' PFS.

	HR (95%CI)	p-value
Ann Arbor Stage	2.33 (0.91-5.98)	0.077
ECOG-PS	2.17 (0.64-7.37)	0.212
Extranodal sites	1.27 (1.12-1.44)	0.0001
β2-microglobulin	1.29 (1.03-1.60)	0.021
5-PS scores	2.09 (0.87-4.99)	0.096
ΔMTV	0.95 (0.63-1.40)	0.820
ΔTLG	0.08 (0.02-0.25)	<0.0001

**Table 8:** Univariate Cox proportional hazards model analysis of ΔMTV, ΔTLG, Clinical Factors and patients' OS.

	HR (95%CI)	p-value
Ann Arbor Stage	2.25 (0.70-7.19)	0.171
ECOG-PS	4.56 (1.25-16.68)	0.021
Extranodal sites	1.36 (1.18-1.57)	<0.0001
β2-microglobulin	1.44 (1.13-1.84)	0.003
5-PS scores	3.92 (1.09-14.09)	0.035
ΔMTV	0.78 (0.51-1.21)	0.281
ΔTLG	0.07 (0.02-0.24)	<0.0001

The optimal cut-off value of ΔTLG is determined by the ROC curve. The best cutoff value was obtained when the AUC of the ROC curve was 0.805. Patients were divided into the ΔTLG≥67.9% group and the ΔTLG<67.9% group; the specificity was 81.2%, while the sensitivity was 71.4%. There were 43 patients in the ΔTLG≥67.9% group and 19 patients in the ΔTLG<67.9% group.

Kaplan–Meier survival curves were plotted based on TLG grouping. Considering the importance of the 5-PS score in evaluating mid-term efficacy and prognosis in patients with DLBCL, we classified patients with scores of 1–3 points as the CR group and patients with scores of 4–5 points as the non-CR group according to the 5-PS score. After classification, there were 31 patients each in the CR and non-CR groups. Kaplan–Meier survival curves were plotted for these groups.

**Figure 4:**
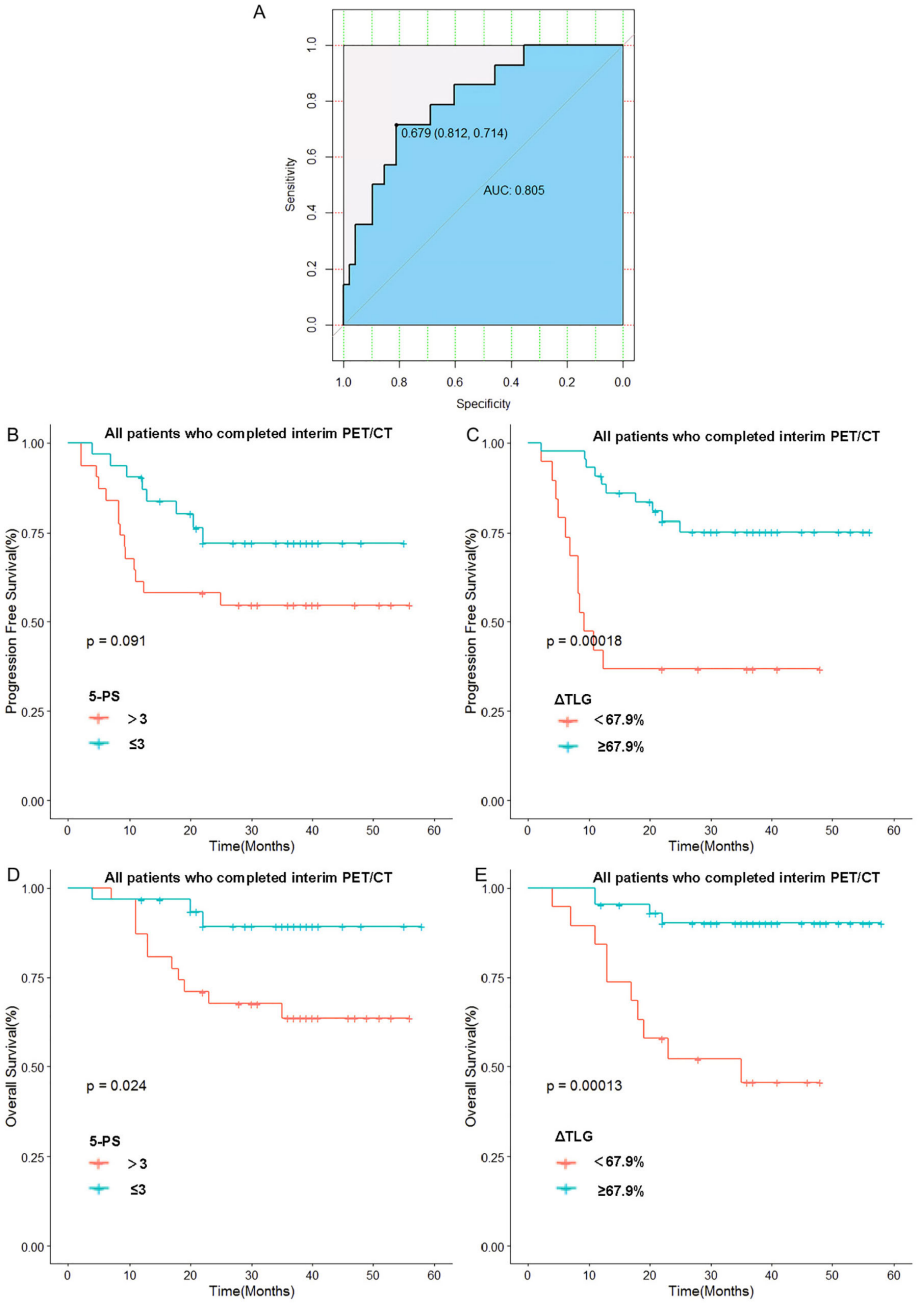
Relationship between 5-PS score, ΔTLG and patients’ prognosis: **(A)** Receiver operating curve of ΔTLG. **(B)** Relationship between 5-PS score and patients’ PFS. **(C)** Relationship between ΔTLG and patients’ PFS. **(D)** Relationship between 5-PS and patients’ OS. **(E)** Relationship between ΔTLG and patients’ OS.

The results showed no statistically significant difference in the 1-year PFS rates between the CR and non-CR groups (P =0.09). However, the 1-year PFS rate of the high ΔTLG group was significantly higher than that of the low ΔTLG group (90.6% vs 42.1%, p=0.0001). Regarding OS, the 1-year OS rate of the CR group was higher than that of the non-CR group (96.7% vs 87.1%, p=0.024), and the 1-year OS rate of the high ΔTLG group was higher than that of the low ΔTLG group (95.3% vs 84.2%, p=0.0001). The results indicate that grouping based on ΔTLG seems to more accurately predict the prognosis of patients with DLBCL compared to grouping based on 5-PS score. **Figure 4** illustrates the associated Kaplan–Meier survival curves.

**Figure 5:**
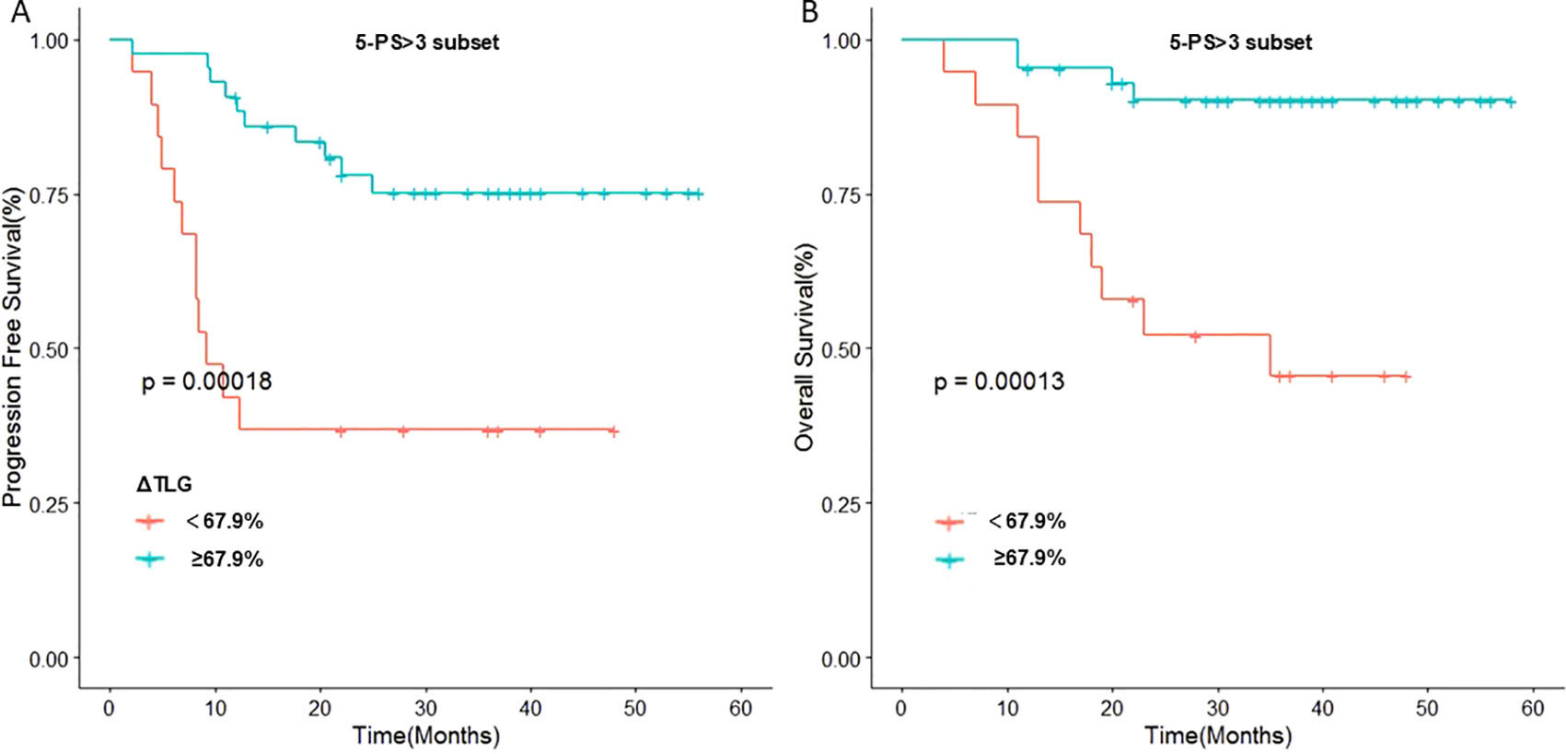
Stratified analysis of 5-PS score: **(A)** Relationship between ΔTLG and PFS in patients with 5-PS score >3. **(B)** Relationship between ΔTLG and OS in patients with 5-PS score >3.

To further verify this result, we used ΔTLG to regroup the patients with 5-PS score of 1–3 points and 4–5 points, respectively. The results showed that among patients with 5-PS score of 1–3 points, there was no significant difference in the 1-year PFS rate (p=0.428) or 1-year OS rate (p=0.428) between high ΔTLG and low ΔTLG groups. However, among patients with 4–5 points, the prognosis of the high ΔTLG and low ΔTLG groups was significantly different. The 1-year PFS rate of high ΔTLG group was significantly higher than that of the low ΔTLG group (82.3% vs 35.7%, p=0.003), and the 1-year OS rate of high ΔTLG group was higher than that of the low ΔTLG group (88.2% vs 85.7%, p = 0.003). **Table 9** shows the specific values, and **Figure 5** illustrates the associated Kaplan–Meier survival curves.

**Table 9:** Univariate Cox proportional hazards model analysis for ΔTLG in patients with a 5-PS score of 1-3.

5-PS score	PFS		OS	
	HR(95%CI)	p-value	HR(95%CI)	p-value
1-3	0.46 (0.09-2.27)	0.337	0.38 (0.03-4.18)	0.428
4-5	0.21 (0.06-0.66)	0.008	0.14 (0.03-0.67)	0.013

**Figure 6:**
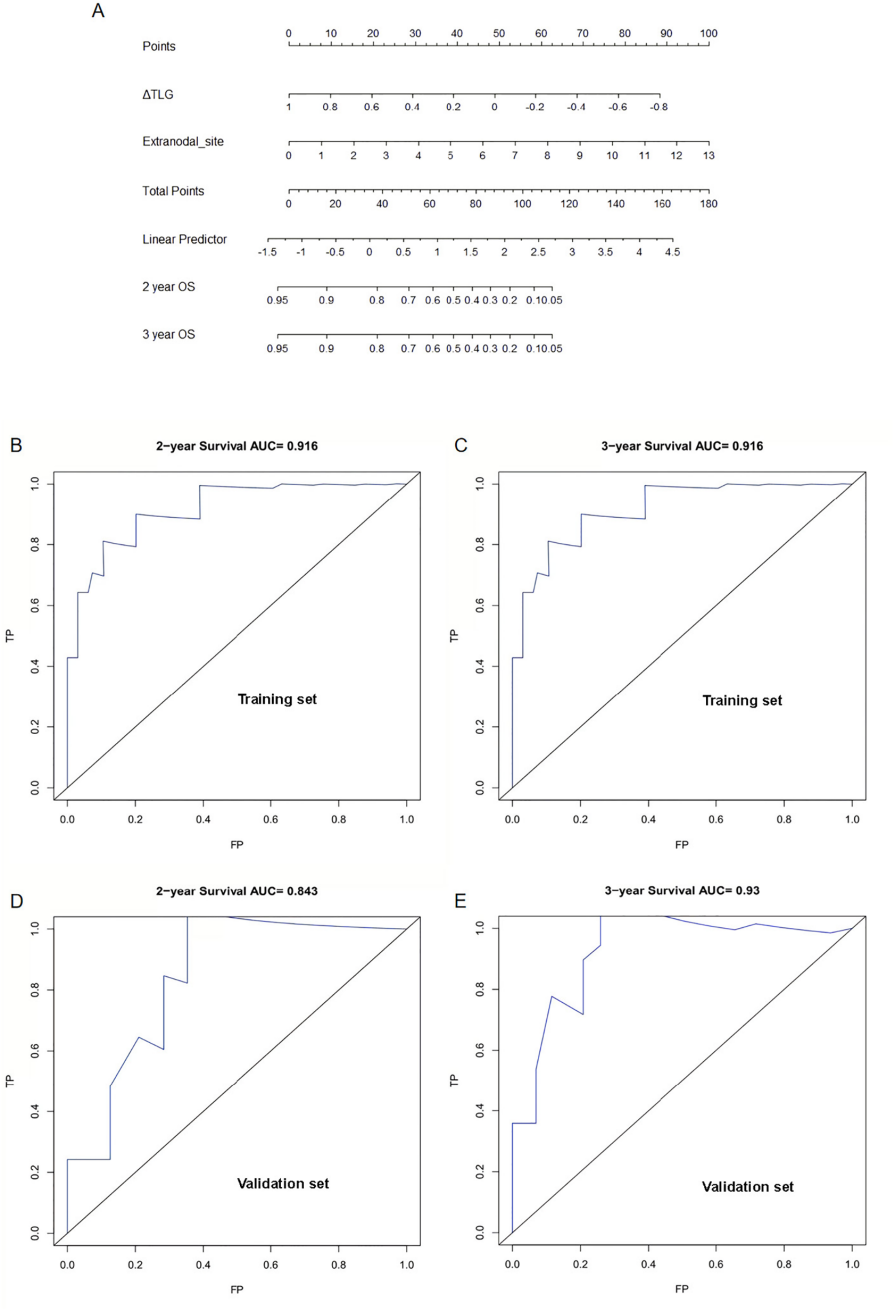
Establishment and verification of nomogram model: **(A)** A Nomogram model for predicting OS in patients with diffuse large B-cell lymphoma by ΔTLG and extranodal sites. **(B)** Area under the curve for predicting 2-year OS in the training set. **(C)** Area under the curve for predicting 3-year OS in the training set. **(D)** Area under the curve predicting 2-year OS in the validation set. **(E)** Area under the curve predicting 3-year OS in the validation set.

Further, 62 patients were randomly divided into the training and validation sets at a ratio of 7:3, resulting in 42 and 20 patients in the training and validation sets, respectively. To perform intergroup comparisons between the training and validation sets, the chi-square test was used for binary variables, and an independent t-test was used for continuous variables. The results, presented in **Table 10**, indicate no statistically significant differences in various indicators between the two groups (28). A nomogram model was developed to predict the OS of patients with DLBCL using the combination of the extranodal sites and ΔTLG. The proportional impact of extranodal sites and ΔTLG on prognosis in the multifactorial Cox proportional hazards model analysis was used to assign scores to each corresponding value for these factors. The scores for each factor are summed to obtain the total score. By analyzing the relationship between the total score and the probability of occurrence of the patient’s outcome event, the odds of 2- and 3-year OS for the respective patients were determined. **Figure 6** shows a specific graph.

**Table 10:** Inter-group Comparison between Training and Validation Sets.

Variable	Total Dataset(N=62)	Training set(N=62)	Validation set(N=62)	p-value
Ann Arbor Stage				0.798
I-II	28	18	10	
III-IV	34	24	10	
ECOG-PS				0.394
0-1	56	37	19	
≥2	6	5	1	
Extranodal sites				0.908
0-1	35	23	12	
≥2	27	19	8	
β2-microglobulin	2.84	2.86	2.73	0.686
5-PS scores				0.853
1-3	31	22	9	
4-5	31	20	11	
ΔTLG				0.233
≥67.9%	43	30	13	
<67.9%	19	12	7	
OS				0.109
Negative Outcome	48	33	15	
Positive Outcome	14	9	5	

The predictive performance of the nomogram model was analyzed, and the C-index of the model was calculated as 0.9. Calibration curves for predicting the 2- and 3-year OS were plotted, and the ROC curves for these calibration curves had an AUC of 0.916 for both. The model was calibrated using the bootstrap method with 1000 iterations for independent sampling.

The model was validated using the same method as for the validation set. The C-index of the model was 0.817. The model was calibrated using the bootstrap method with 1000 iterations for independent sampling. Calibration curves for predicting 2- and 3-year OS were plotted, and the ROC curves for these calibration curves had AUCs of 0.843 and 0.93, respectively. The results indicated that the calibration curves had good predictive performance. We also plotted calibration curves for the model, as shown in **Supplementary Figure S2**.

#### 3.2.2 Presentation of real cases

**Figure 7:**
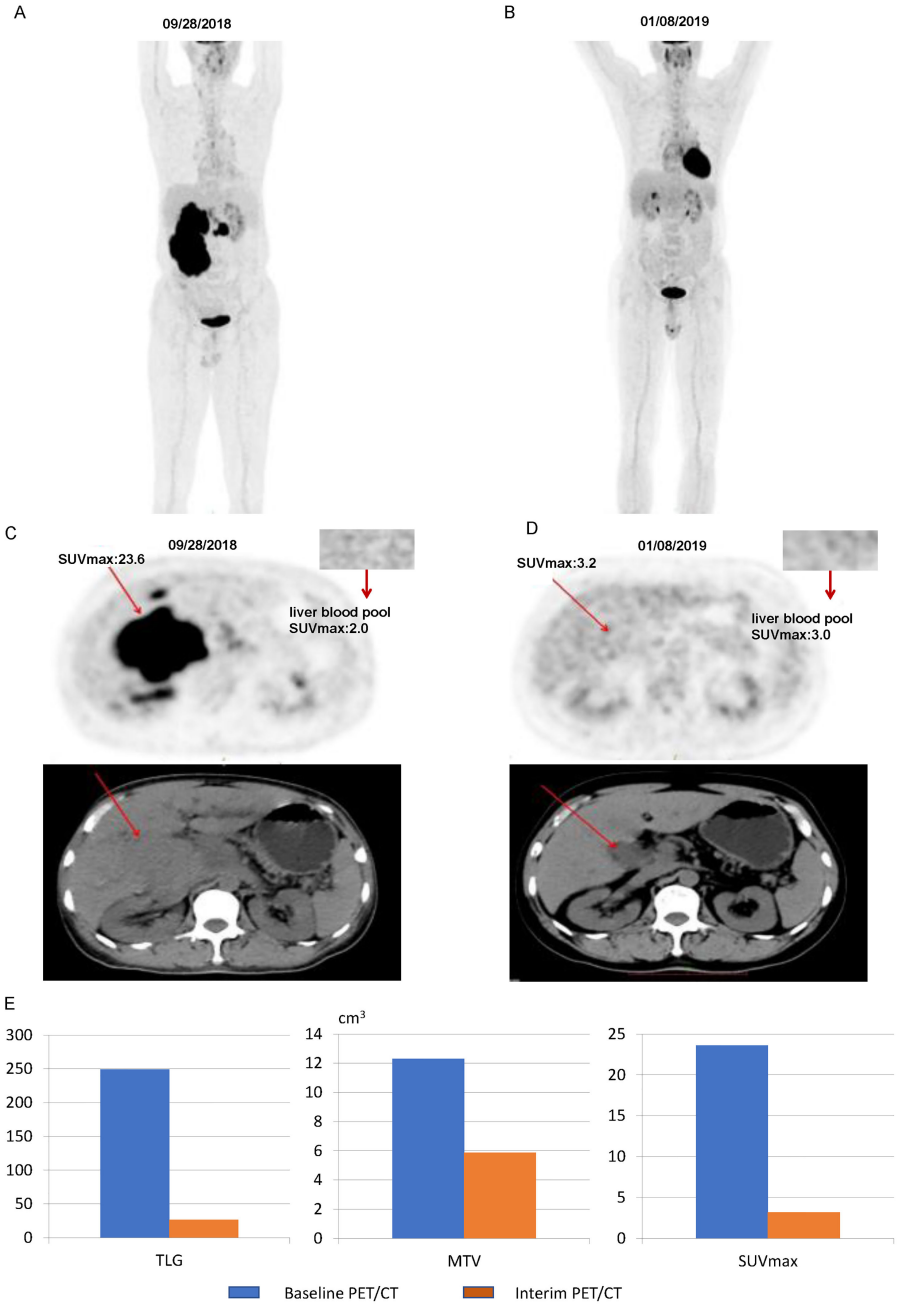
Images of a patient with a 5-PS score of 4: **(A)** Baseline PET/CT images of the whole body. **(B)** Interim PET/CT images of the whole body. **(C)** Baseline PET/CT images of the lesion. **(D)** Interim PET/CT images of the lesion. **(E)** Changes in TLG, MTV, and SUVmax at mid-term efficacy evaluation.

**Figure 8:**
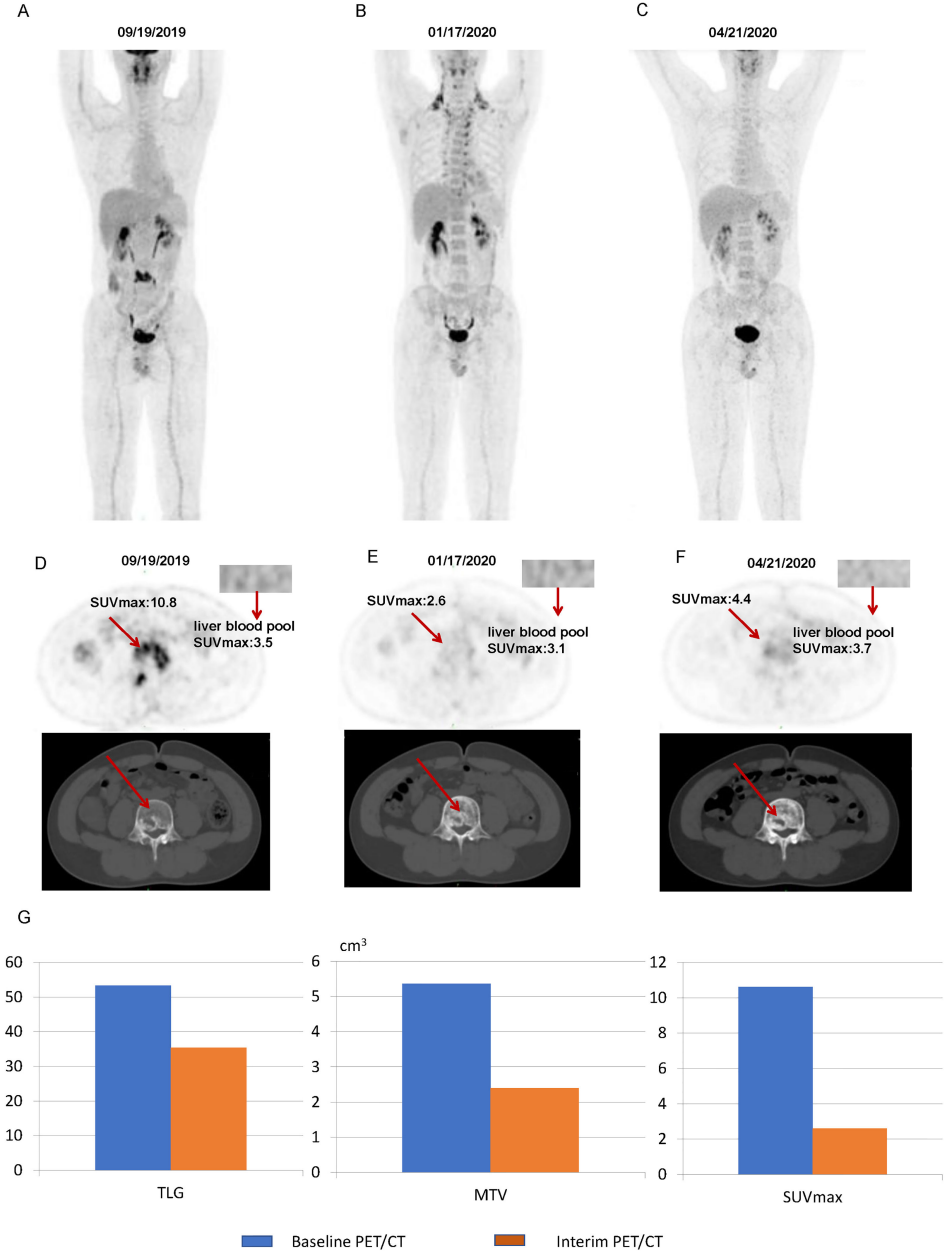
Images of a patient with a 5-PS score of 3: **(A)** Baseline PET/CT images of the whole body. **(B)** Interim PET/CT images of the whole body. **(C)** Post-treatment PET/CT images of the whole body. **(D)** Baseline PET/CT images of the lesion. **(E)** Interim PET/CT images of the lesion. **(F)** Post-treatment PET/CT images of the lesion. **(G)** Changes in TLG, MTV, and SUVmax at mid-term efficacy evaluation.

**Figure 7** illustrates some imaging findings in a 60-year-old male patient with DLBCL. The patient underwent baseline PET/CT prior to treatment, and the results showed multiple tumors in the porta hepatis area and abdomen; the larger ones were approximately 12.2 × 5.8 × 17.2 cm, SUVmax: 23.6. The patient subsequently received three cycles of the R-CHOP regimen. Interim PET/CT showed that the original tumor had shrunk to approximately 3.2 × 3.6 cm, SUVmax: 3.2. The patient’s 5-PS score was 4 points. According to the standard 5-PS score, the patient’s prognosis may be poor. Different from the results obtained using the 5-PS score, the patient’s ΔTLG was calculated to be 89.4% using PET/CT Lesion Quantifier. According to the standard of ΔTLG, the prognosis of patients may be better. The patient survived for 60 months. **Figure 8** illustrates some of the imaging findings of a 28-year-old male patient with DLBCL. Baseline PET/CT showed bone destruction in the L4 vertebral body with an SUVmax of 10.8. The patient subsequently received three cycles of RCHOP treatment. The interim PET/CT showed that bone destruction of the L4 vertebral body was essentially the same as before (SUVmax: 2.6). The patient’s 5-PS score was 3. According to the 5-PS score standard, this patient may have a good prognosis. Different from the results obtained using the 5-PS score method, the patient’s ΔTLG was calculated to be 33.6% using PET/CT Lesion Quantifier. According to the standard of ΔTLG, the patient’s prognosis may be poor. In fact, the patient’s condition relapsed after 6.9 months of treatment.

## 4 Discussion

In DLBCL, whether SUVmax is an independent prognostic factor remains controversial. However, previous studies have shown that SUVmax is an independent prognostic factor (29, 30). Recently, with an increased understanding of volumetric metabolic parameters and observations from large samples, researchers have concluded that volumetric metabolic parameters can improve the accuracy of DLBCL predictions (22). They argued that neither SUVmax nor SUVmean could effectively predict the treatment response, PFS, and OS (20). Ceriani et al. (18) extensively studied whether SUVmax could serve as a predictor of PFS and OS in a DLBCL population, including 141 patients in a training cohort and 113 patients in a validation cohort, both with similar Ann Arbor stages. In both cohorts, SUVmax was not a significant predictor of PFS or OS. In a larger study evaluating 169 patients with DLBCL treated with R-CHOP (Ann Arbor stages II and III without extranodal lesions), Song et al. (20) found that patients with an MTV <220 cm^3^ had significantly better PFS and OS. Even after multivariate Cox regression analysis for stages II and III disease, the correlation between MTV and prognosis remained significant. Therefore, they concluded that the MTV is an independent predictor of PFS and OS in patients with DLBCL, regardless of the Ann Arbor stage. However, in a study involving 91 patients, Zhou et al. (31) reported that despite the association of high baseline MTV and TLG with poor prognosis, only TLG was an independent predictor of PFS and OS. In this study, patients with high TLG levels were more prone to relapse during treatment, even if they achieved complete remission, compared with patients with low TLG levels (40% vs. 9%, p = 0.012). Our study utilized the third quartile of liver uptake values as the threshold to measure MTV and TLG, exploring the relationship between MTV, TLG, and the prognosis of patients with DLBCL. The results of the univariate Cox proportional hazards model analysis indicated that the SUVmax did not exhibit statistical significance in relation to either PFS or OS in the DLBCL population in this study. This could be attributed to the sensitivity of SUVmax being influenced by various factors, such as the time interval between injection and scanning, partial volume effects in small lesions, attenuation of tracer activity, technical characteristics, acquisition, and reconstruction parameters of the scanner. Additionally, SUVmax only records the intensity of ^18^F-FDG uptake in the most metabolically active region, making it challenging to reflect the overall tumor burden of patients, especially in cases of DLBCL where multiple lesions are common, leading to a larger tumor burden. Therefore, the accuracy of predicting prognosis based on the SUVmax measured from the most intense lesion before treatment has significant limitations.

In our study, although the MTV showed a significant correlation in the univariate analysis, the results of the multivariate analysis showed that only the TLG was an independent predictor of patient prognosis. In terms of calculation methods, MTV and TLG showed a certain correlation; however, a significant difference was found in this study between MTV and TLG in predicting the OS and PFS of patients with DLBCL. This difference may be related to the definitions of the MTV and TLG. MTV represents the volume of all pixels on PET images that exceed a preset SUV value based on the assumption of elevated metabolism in tumors than in normal tissues. On the contrary, TLG is a metabolic parameter derived by multiplying MTV with SUVmean. These MTV results, to some extent, overlook the intensity of tumor metabolism; thus, failing to accurately reflect the overall tumor burden, especially in lesions with non-uniform metabolism where differences might be more pronounced. In contrast, TLG not only reflects the metabolic activity of the tumor, but also considers the metabolic volume, aligning more closely with the principles of PET imaging and the concept of tumor burden. This more reliably reflects the patient’s tumor burden. Therefore, we believe that the TLG, relative to the MTV, is a more reliable indicator for predicting the prognosis of patients with DLBCL.

Compared with Ann Arbor staging, TLG seems to have an advantage in predicting patient prognosis. In patients with stage I-II, the 1-year PFS rate and 1-year OS rate of patients in the low TLG group were significantly higher than those in the high TLG group. The same results were found in patients with stage III-IV. This indicates that in patients with stages I–II disease, the tumor burden of some patients might be underestimated. Although these patients have fewer lymph nodes or organs involved, their tumor volume is larger, or their metabolism is more active, resulting in a larger tumor burden and relatively poorer treatment outcomes. Similarly, in patients with stage III–IV disease, the tumor burden of some patients might be overestimated. Although these patients have more lymph nodes or organs involved, their tumor volume is smaller, or their metabolism is less active, resulting in a smaller tumor burden and better treatment outcomes. By calculating TLG, it is possible to distinguish between these patients, formulate individualized treatment plans, and improve their prognosis. For example, for patients with Ann Arbor stage I or II disease but with high TLG levels, we can consider adding radiation therapy appropriately after completing all cycles of R-CHOP treatment. However, this hypothesis must be tested in a larger study.

Although some studies (32, 33) have found that interim PET/CT is not a prognostic factor for patients with DLBCL, others (34–36) have indicated that interim ^18^F-FDG PET/CT is an effective predictor of survival in patients with DLBCL. In our study, the high ΔTLG group had a higher 1-year OS rate than the low ΔTLG group, indicating that a higher ΔTLG is associated with a better prognosis in patients with DLBCL. The interim PET/CT examination was conducted after the third or fourth cycles of R-CHOP or R-CHOP modified regimen treatment to reduce the impact of false positives, we found that high ΔTLG is an independent predictor of favorable PFS and OS in patients with DLBCL. This implies that after 3–4 cycles of R-CHOP treatment, patients with DLBCL can be assessed for treatment effectiveness and prognosis by detecting changes in TLG parameters, allowing for timely adjustment of treatment plans, especially for patients with a possible poor prognosis. Admittedly, the baseline and interim PET/CT scans in this study were from the same group of patients, and it is possible that the same patient could draw different conclusions from the two analyses. We believe that a small number of patients with a higher tumor burden can still obtain a better prognosis if they are sensitive to treatment options. The TLG of baseline PET/CT may be more informative for the staging of DLBCL patients and the development of the initial treatment regimen, while the ΔTLG may be able to monitor the sensitivity of patients to the treatment regimen. For patients who are not responsive to first-line regimens, the treatment plan may be adjusted in advance to prolong their survival. For patients who originally needed maintenance treatment, if the baseline PET/CT shows that the tumor burden is small, and the interim PET/CT shows that they are sensitive to treatment, we can consider not performing maintenance treatment after completing the entire cycle of treatment. However, this hypothesis must be verified in larger studies.

Compared with 5-PS score, ΔTLG also has a significant correlation with a patient’s prognosis. Especially in terms of observing the long-term survival of patients, ΔTLG seems to have more advantages. Patients with a 5-PS score >3 are usually considered to have a poor prognosis. Therefore, we grouped patients with a 5-PS score >3 points again according to ΔTLG. It was found that among patients with a 5-PS score >3 points, there is still a significant difference in the prognosis between the high ΔTLG group and the low ΔTLG group. Based on these results, we considered that some patients with a 5-PS score >3 achieved long-term survival. As in the cases illustrated in **Figures 7** and **8**, clinicians can often encounter situations in which a portion of patients with a 5-PS score of ≤3 have a poor prognosis, whereas a portion of patients with a 5-PS score of >3 have a long**-**term survival, which may be due to the fact that PET/CT has some false negative and false positive rates. Although the negative predictive value of interim PET/CT is high (>80%), the positive predictive value is significantly lower (approximately 15%), resulting in a greater prognostic variability in patients with a 5-PS score >3. This is because of the inability of interim PET/CT to distinguish between the presence of residual surviving tumor tissue and a nonspecific inflammatory host response (12, 13). In our study, the prognostic difference between the high ΔTLG group and the low ΔTLG group was not statistically significant in patients with a 5-PS <3 score. This finding also suggests that the negative predictive value of interim PET/CT is high. However, ΔTLG does not depend on a single tumor tissue, it is based on the patient’s own liver uptake value to measure TLG. This can minimize the error caused by the false positive rate of PET/CT, which can help to screen out patients with a 5-PS score >3 points but a good prognosis. Moreover, the 5-PS score is subject to human subjectivity, and different physicians may score the same patient differently, whereas the TLG based on PET/CT Lesion Quantifier measurements significantly reduces the influence of human subjective consciousness on the results. The limitations of this study are that it was a single-center retrospective study with a small number of patients included, and the conclusions drawn need to be validated in a larger study. Nevertheless, the data in this study illustrate that ΔTLG may be another reliable indicator of interim efficacy assessment, in addition to the 5-PS score.5. We did not split the baseline PETCT data into control and experimental groups, but we did so for the interim PETCT data. This is because once again there have been many pre-vious studies demonstrating the role of baseline TLG in predicting survival in patients with non-Hodgkin’s lymphoma, including DLBCL (37–39), whereas there have been few studies on the relationship between ΔTLG and survival in patients with DLBCL, which is one of our innovations. In most patients, the results of baseline TLG and ΔTLG are not contradictory, that is, patients with high baseline TLG are more likely to have a smaller ΔTLG. We do not advocate direct comparison of these two parameters because baseline TLG tends to stratify patients before treatment, while ΔTLG mainly assesses whether the patient is sensitive to first-line treatment. But as you said, there are indeed a few patients with relatively large baseline TLG and relatively large ΔTLG. This small number of cases shows that although a few patients have a large tumor burden, they are very sensitive to the treatment regimen. These patients may have a good prognosis. In this process, ΔTLG plays a role of re-evaluation. At present, the treatment of DLBCL has entered the era of R-CHOP+X (new drugs of different types). ΔTLG may be able to quantify the efficacy of new therapies, making the short-term efficacy of different innovative therapies more comparable. We provide a case in the **Supplementary Material** that may help understand their relationship (case.docx).

## 5 Limitations

This study has several limitations. First, it is a retrospective analysis, which may introduce selection bias. Second, the sample size is relatively small, limiting the generalizability and statistical significance of the findings. Additionally, the lack of external validation may affect the reliability of our results. Despite these limitations, this study demonstrates a strong cor-relation between ΔTLG and patient's prognosis, highlighting the significant potential of in-terim PET/CT in assessing patient's prognosis. These findings provide an important basis for future clinical applications.

## 6 Conclusions

Baseline TLG may be able to distinguish patients with poor prognosis among those with Ann Arbor staging of stage I-II. Moreover, ΔTLG may distinguish patients with good prognosis among those with 5-PS score >3. TLG will hopefully help clinicians develop more individualized treatment plans and improve the prognosis of DLBCL patients, and we call on more scholars to devote themselves to the study of metabolic parameters of PET/CT.

## Data Availability

The original contributions presented in the study are included in the article/**Supplementary Material**. Further inquiries can be directed to the corresponding author.
