# Supplementary material for: Baseline and interim 18F-FDG PET/CT metabolic parameters predict the efficacy and survival in patients with diffuse large B-cell lymphoma
Source: Front Oncol. 2024 Oct 7;14:1395824. doi: 10.3389/fonc.2024.1395824 (PMC11491437; doi:10.3389/fonc.2024.1395824)
Supplement: Supplementary file 1 [file DataSheet1.docx]

**Supplementary Material**

Inspired by the Deauville score, which uses mediastinal and liver uptake values as scoring criteria, we used the mean, third quartile, maximum, 1.5 times the mean, and 2 times the mean of mediastinal and liver uptake values as thresholds to measure MTV and TLG, respectively. Moreover, the relationship between MTV and TLG measured at different thresholds and the prognosis of patients with diffuse large B-cell lymphoma was explored. As the mediastinum is not a solid organ, blood flow will significantly affect its uptake values, which will lead to excessive differences in the uptake values at different layers. To minimize errors, the three levels of the mediastinum were boxed separately, and the uptake values at the three levels of the mediastinum were derived and averaged to set the thresholds. Univariate and multivariate regression analyses were performed using the Cox proportional risk model to evaluate the relationship between ^18^F-FDG PET/CT metabolic parameters and the patients' PFS and OS and to screen out the parameters that had the most significant relationship with the prognosis of patients with DLBCL. To better demonstrate the Hazard Ratio (HR) and 95% Confidence Interval (95% CI) in the Cox proportional risk model, PET/CT metabolic parameters were scaled down exponentially (100-fold for MTV and 1000-fold for TLG) prior to analysis using the Cox proportional risk model. The results are presented in Tables 1 – 4.

**Supplementary Table 1.** Relationship between MTV,TLG that was measured using mediastinal uptake values as threshold values, and patients' PFS

|  | 95% CI | P-value |
| --- | --- | --- |
| MTV _mean_ | 1.23(1.00–1.50) | 0.05 |
| MTV _third quartile_ | 1.22(0.95–1.50) | 0.07 |
| MTV _maximum_ | 1.21(0.90–1.23) | 0.20 |
| MTV _1.5 times the mean_ | 1.21(0.93–1.56) | 0.15 |
| MTV _2 times the mean_ | 1.21(0.86–1.69) | 0.27 |
| TLG _mean_ | 1.20(0.90–1.60) | 0.21 |
| TLG _third quartile_ | 1.20(0.89–1.62) | 0.22 |
| TLG _maximum_ | 1.21(0.85–1.72) | 0.28 |
| TLG _1.5 times the mean_ | 1.21(0.88–1.66) | 0.24 |
| TLG _2 times the mean_ | 1.18(0.82–1.70) | 0.36 |

**Supplementary Table 2.** Relationship between MTV and TLG, which were measured using mediastinal uptake values as threshold values, and the patients’ OS

|  | 95%CI | P-value |
| --- | --- | --- |
| MTV _mean_ | 1.28(1.02–1.61) | 0.03 |
| MTV _third quartile_ | 1.26(0.99–1.60) | 0.06 |
| MTV _maximum_ | 1.15(0.81–1.64) | 0.44 |
| MTV _1.5 times the mean_ | 1.21(0.90–1.64) | 0.21 |
| MTV _2 times the mean_ | 1.16(0.77–1.74) | 0.47 |
| TLG _mean_ | 1.39(1.01–1.90) | 0.04 |
| TLG _third quartile_ | 1.37(0.99–1.90) | 0.06 |
| TLG _maximum_ | 1.21(0.80–1.83) | 0.37 |
| TLG _1.5 times the mean_ | 1.27(0.89–1.83) | 0.19 |
| TLG _2 times the mean_ | 1.17(0.75–1.82) | 0.47 |

**Supplementary Table 3.** Relationship between MTV and TLG, which were measured using liver uptake values as threshold values, and the patients’ PFS

|  | 95%CI | P-value |
| --- | --- | --- |
| MTV _mean_ | 1.31(1.10–1.56) | 0.003 |
| MTV _third quartile_ | 1.30(1.10–1.59) | 0.003 |
| MTV _maximum_ | 1.37(1.07–1.75) | 0.012 |
| MTV _1.5 times the mean_ | 1.35(1.05–1.70) | 0.009 |
| MTV _2 times the mean_ | 1.33(0.98–1.80) | 0.066 |
| TLG _mean_ | 1.58(1.20–2.10) | 0.001 |
| TLG _third quartile_ | 1.68(1.25–2.26) | <0.001 |
| TLG _maximum_ | 1.70(1.23–2.34) | 0.001 |
| TLG _1.5 times the mean_ | 1.05(1.01–1.08) | 0.006 |
| TLG _2 times the mean_ | 1.61(1.10–2.35) | 0.01 |

**Supplementary Table 4.** Relationship between MTV and TLG, which were measured using liver uptake values as threshold values, and the patients’ OS

|  | 95%CI | P-value |
| --- | --- | --- |
| MTV _mean_ | 1.38(1.15–1.66) | <0.001 |
| MTV _third quartile_ | 1.40(1.15–1.69) | <0.001 |
| MTV _maximum_ | 1.47(1.14–1.89) | 0.003 |
| MTV _1.5 times the mean_ | 1.44(1.13–1.82) | 0.002 |
| MTV _2 times the mean_ | 1.43(1.05–1.95) | 0.02 |
| TLG _mean_ | 1.73(1.28–2.34) | <0.001 |
| TLG _third quartile_ | 1.83(1.33–2.50) | <0.001 |
| TLG _maximum_ | 1.84(1.31–2.59) | <0.001 |
| TLG _1.5 times the mean_ | 1.06(1.02–1.10) | 0.001 |
| TLG _2 times the mean_ | 1.79(1.20–2.66) | 0.004 |

From the results, we can see that the vast majority of MTV and TLG measured with the mediastinal uptake value as the threshold showed no significant correlation with the patients' PFS and OS. This may be related to the fact that the mediastinum is not a solid organ, and its blood flow distribution is not homogeneous. Moreover, it is easily affected by factors, such as contrast injection and examination time, which resulted in the patient's uptake value on different layers of the mediastinum varying too much. The MTV and TLG measured with the liver uptake value as the threshold showed a significant correlation with the patients' PFS and OS, among which the TLG measured in the third quartile of the liver uptake values was the most significant (PFS: p<0.001, OS: p <0.001); therefore, we chose the third quartile of the liver uptake values as the threshold for the measurement of MTV and TLG in patients for the next step of the study.

In view of the significant correlation between MTV and patient prognosis in the univariate Cox proportional hazards model analysis, we divided the patients into the MTV ≥ 17.468 group and the MTV < 17.468 group and drew the Kaplan–Meier curve.

**Supplementary Figure 1**. Relationship between MTV and the patients‘ PFS and OS.


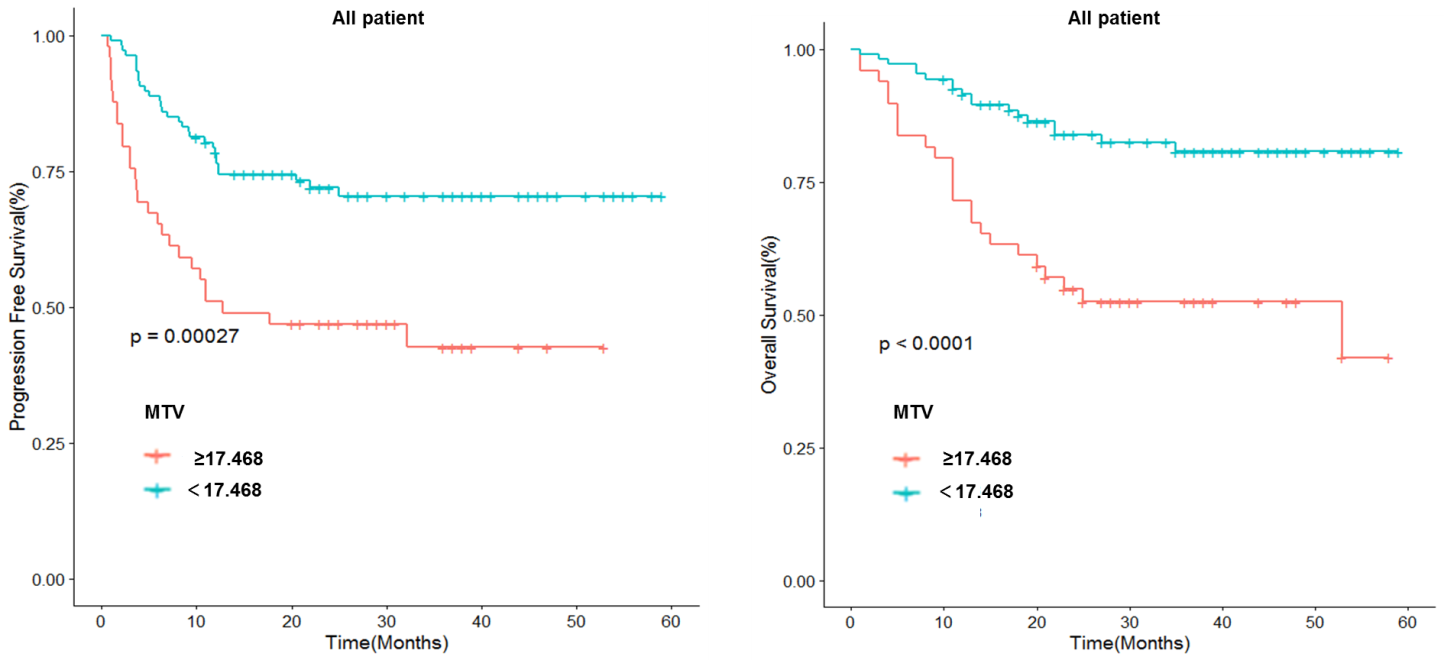


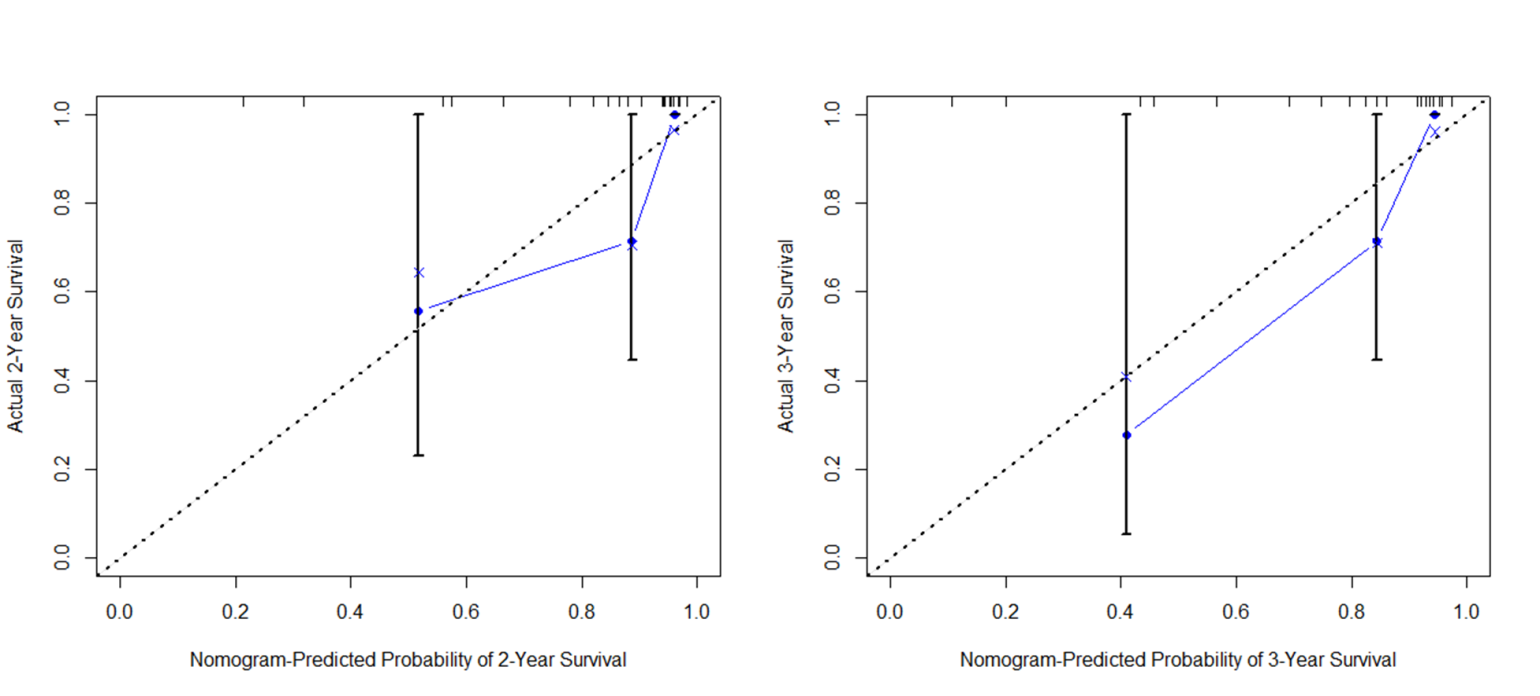
**Supplementary Figure 2**. Calibration curves for predicting the patients' 2-year OS and 3-year OS.
